# Platelet counts and mean platelet volume in association with serum magnesium in maintenance hemodialysis patients

**DOI:** 10.12861/jrip.2012.08

**Published:** 2012-01-01

**Authors:** Mahmoud Rafieian-Kopaie, Hamid Nasri

**Affiliations:** ^1^Medical Plants Research Center, Shahrekord University of Medical Sciences, Shahrekord, Iran; ^2^Department of Nephrology, Isfahan University of Medical Sciences, Isfahan, Iran

**Keywords:** Platelets, Magnesium, End-stage renal disease, Mean platelet volume

## Abstract

**Introduction:** Platelet dysfunction is responsible for increased bleeding tendency of chronic renal failure patients. Magnesium (Mg) retention can occur in patients on maintenance hemodialysis.Objectives: Studies concerning the impact of magnesium disturbances on platelet counts and mean volume in hemodialysis are quiet scarce.

** Patients and Methods:** A total of 36 (f= 15, m= 21), HD patients were included. The mean patients’ age was 46 (±16) years. The mean length of time patients had received hemodialysis was 32 (±36) (median: 19) months.

** Results:** The mean platelet count was 165 (±70) (median: 163) [x10^3^μ/L]. The mean platelet volume was 9 (±1) (median: 9.2) fl. The mean Mg was 9.2 ±1.4 (median: 2.4) mg/dl. In this study a significant inverse correlation of platelet count with mean platelet volume (r= -0.39, p= 0.017) was seen. A significant inverse correlation of serum Magnesium with mean platelet volume (r= -0.37, p= 0.025) was seen. There was not significant association of serum Mg and PLT count was found too (p> 0.05).

**Conclusion:** A significant inverse correlation of serum Magnesium with mean platelet volume needs further investigations to clarify the clinical significance of this finding.

**Implication for health policy/practice/research/medical:**

Platelet dysfunction is responsible for increased bleeding tendency of chronic renal failure patients. Magnesium retention can occur in patients on maintenance hemodialysis. In a study on 36 chronic hemodialysis patients, we found a significant inverse correlation of serum magnesium with mean platelet volume. This finding needs further investigations to clarify its clinical significance.

## Introduction

End-stage renal disease patients mostly suffer from various hemostatic disorders (1). Bleeding tendency in uremic patients is high, due to abnormalities of primary hemostasis (1,2). This is particularly attributed to platelet dysfunction (1,2). In this regard, the most important abnormalities are related to decreased availability of platelet factor-3, defective platelet aggregation, adhesiveness, and prolongation of the bleeding time (2,3). The implicated mechanisms include: increased vessel wall prostacyclin, abnormal platelet arachidonic acid, increased levels of parathyroid hormone, platelet (PLT) inhibition by various plasma metabolites, such as urea, guanidinosuccinic acid, and phenolic acid metabolism (1-3). Regarding the PLT count, hematologic analyzers have introduced a reliable index. In association with the platelet parameter, the mean platelet volume (MPV) has been described (2-4). MPV is a determinant and a relatively a good marker for PLT function in that large platelets are more active than normal sized ones. Mean platelet volume, which is considered as a measure of platelet size, reflects changes in either the rate of platelet production or the level of platelet stimulation (3-6).

Increased mean platelet volume may indicate increased numbers of large, hyperaggregable platelets or increased platelet activation which is accepted as an independent coronary artery risk factor (3-8). In general population, MPV is usually considered as an independent risk factor for myocardial infarction (MI) and coronary heart disease in hemodialysis (HD) patients (4,5).

Magnesium (Mg) is the eight most common elements in the crust of the Earth (6-8). It is an important intracellular cation which is distributed into three major compartments: mineral phase of bones (65%), intracellular space (34%) and extracellular fluid (1%) (6-8). In HD patients, both total and ionized magnesium levels are often slightly elevated above the normal range and have been shown to be dependent on residual renal function (6-10). Mg has various physiological functions in the body in health and in diseases. Mg retention can occur in patients on maintenance HD (10-13).

## Objectives

Regarding the present data, studies regarding the association of magnesium disturbances in maintenance HD with platelet counts and MPV are quit scarce. We therefore aimed to elucidate whether in patients with uremia on hemodialysis, the level of plasma Magnesium affects the mean platelet volume and count.

## Patients and Methods

### Patients

The etiologies of renal failure were diabetic nephropathy, hypertension, various glomerular diseases, autosomal dominant poly cystic kidney disease (ADPKD) and also urinary tract infections . Due to the severity of secondary hyperparathyroidism, each patient was treated with calcium carbonate capsules, and Rena-Gel (sevelamer; Genzyme Europe B.V.; United Kingdom/Ireland) capsules and vitamin D3 tablets (Calcitriol; Rocaltrol) (Roche Hexagon; Roche Laboratories Inc, New Jersey, USA), at various doses. Due to the severity of anemia, patients were received iron sucrose, (Venofer -International Inc. St. Gallen Switzerland) intravenously, at various doses after each dialysis session . Patients were also administered 6 mg/day/day folic acid, 500 mg/day Acetyl-L-Carnitine (Jarrow Formulas, Inc™ Los Angeles, CA), 2,000 U intravenous Eprex (recombinant human erythropoietin [Rhuepo] [Janssen-Cilag; Cilag - AG International 6300 Zug, Switzerland) and one vitamin B-complex tablet/day after each dialysis session. Exclusion criteria were any active or chronic infection and use of angiotensin converting enzyme inhibitors, Non-steroidal anti-inflammatory drugs or the use of other drugs having adverse effects on platelet production or function.

### Laboratory methods

Blood samples were collected from patients after an overnight fasting. Complete blood count containing mean platelet volume (MPV) (Ref. Range 7.5-11.5 fl) and platelet count were measured using a Sysmex-KX-21N cell counter (Sysmex Corporation; Mundelein, Illinois, Sysmex America, Inc.). Level of serum magnesium (Mg), calcium (Ca), phosphorus (P) and also alkaline phosphatase (ALP) were measured using standard kits. Duration and dosages of HD treatment were calculated from the patients’ records. The duration of each HD session was 4 hours. For patients predialysis creatinine, BUN and post dialysis BUN were measured. The urea reduction ratio was calculated using the formula 100 × (1-[C_t_/C_o_]), in which Co is the predialysis blood urea nitrogen and C_t_ is the blood urea nitrogen measured five minutes after the end of dialysis.

### Ethical issues

(1) The research followed the tenets of the Declaration of Helsinki; (2) informed consent was obtained; (3) the research was approved by the institutional review board.

### Statistical analysis

Statistical correlations were evaluated using the partial correlation test. Results are expressed as means±SD. Statistical analyses were done using SPSS 11.5 (SPSS Inc, Chicago, IL). P less than 0.05 was considered as statistically significant.

## Results

**Figure 1:**
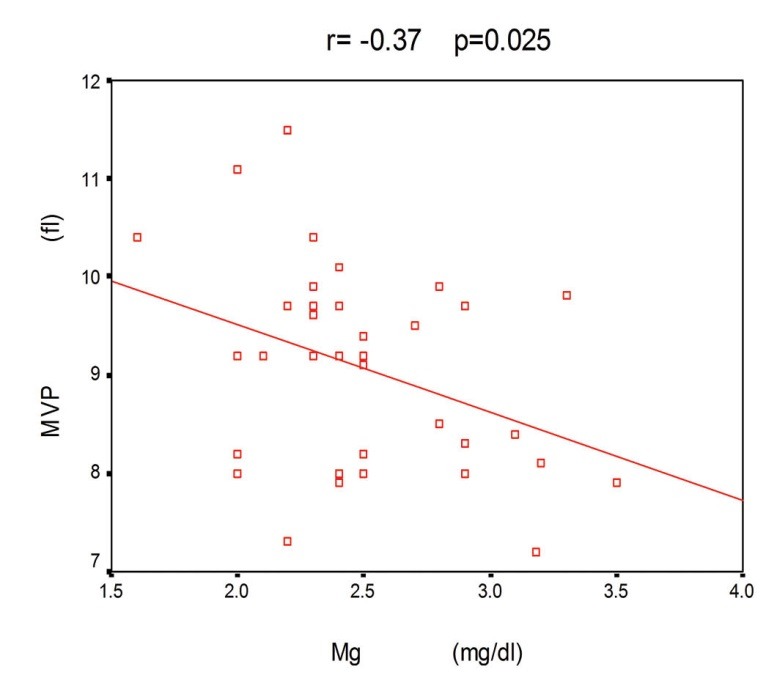
Significant inverse association of serum Mg with MPV.

The total patients were 36 (f= 15, m= 21). The mean patients’ age was 46 (±16) years. The mean length of time patients had received HD was 32 (±36) (median: 19) months. The mean PLT count was 165 (±70) (median: 163) [x10^3^µ/L]. The mean MPV was 9 (±1) (median: 9.2) fl. The mean Mg was 9.2±1.4 (median: 2.4) mg/dl. Table 1, summarized patients’ data. In this study a significant inverse correlation of platelet count with mean platelet volume (r= -0.39, p= 0.017) was seen. A significant inverse correlation of serum Mg with MPV (r= -0.37, p= 0.025; Figure 1) was seen. There was not significant association of serum Mg and PLT count was found (p> 0.05).

**Table 1:** Mean±SD, minimum and maximum of age, duration and doses of hemodialysis and also laboratory results of patients

**Total patients=36**	**Minimum**	**Maximum**	**Mean**
Age years	16	80	46±16
DH* months	2	156	32± 36
Dialysis	36	1584	294± 393
Dose sessions			
URR%	39	76	59± 9
Creat mg/dl	3	18	9± 3
BUN mg/dl	30	180	82± 33
Ca mg/dl	5	10	7.6± 0.9
Alb g/dl	24	4.8	3.8± 0.5
PLT count [x10^3^µ/L]	264	396	165± 70
MPV (fl)	7	11	9± 1
Hgb g/dl	5	13	9± 2
HCT%	14	40	28± 6
*Duration of hemodialysis treatment			

## Discussion

In this study we found a significant inverse correlation of PLT count with mean platelet volume. A significant inverse correlation of serum Mg with MPV was also found. The greatest role in the development of haemostatic disturbances in HD patients is ascribed to the platelets (14). Other than platelet count, the mean platelet volume is used by clinician, however, its role in the diagnosis and management of patients is unclear. While factors which during HD affect volume PLT and count are under investigation, it is believed that platelet aggregation and activation, and coagulative activation are the most important and earliest phenomena that occur after contact between artificial membranes and blood (15,16). Mean platelet volume is a physiological factor of hemostatic importance (15-17). Large platelets are more reactive and aggregate more easily (16-18). Furthermore, they contain more dense granules and release more serotonin in comparison to small platelets (16-19). There are not any nuclei in platelets and their characteristics are evaluated by their progenitor cells, the bone marrow megakaryocytes (16-20). The platelet density and volume are determined at thrombopoiesis and when in the circulation, platelets do not change in size (21-23). The mechanisms that control the platelet production are not fully understood, although it has been suggested that both platelet counts and MPV are under independent hormonal control (23,24). It should be noted that larger platelets are more reactive (23,24). The inverse association of platelet count with mean platelet volume, shown in our study, was also shown in the studies conducted by others (25,26). An inverse correlation between platelet number and platelet volume has been reported in 564 normal subjects and 297 pregnant women (26). In our previous study regarding association of C-reactive protein with PLT count and MPV in HD patients, we found a significant inverse correlation of MPV with serum CRP (27). In this study no significant correlation of PLT count and serum CRP was found (p>0.05) (28). In another study on the same patients, we found a significant positive association between PLT count and plasma HCO_3_^-^ and also a significant inverse correlation of MPV with plasma HCO_3_^-^ were found (28). To understand the impact of parathormone on platelet count and MPV in HD patients, we also conducted a study in which we found, a near significant positive correlation between the mean platelet volume and serum parathormone. A significant inverse correlation between platelet count and serum parathormone was also seen (29). Magnesium ion (Mg^2+^) is found to play an important role in cell activation (28-31). It was found that, Mg^2+^ deficiency is associated with platelet hyper-reactivity (28-31). Previous studies have shown reduced PLT release of β-thromboglobulin and thromboxane B_2 _with increasing magnesium concentrations (0.5–8.0 mmol/l) (30-33). In addition, magnesium has also been shown to reduce thrombin-stimulated Ca^+2^ influx in platelets (30-33). Magnesium has been shown to reduce platelet aggregation both in vitro and ex vivo (30-34). To the best of our knowledge, this is the first report concerning association of platelets count and mean volume with serum Mg in HD patients. Hence, further studies are needed to clarify the clinical significance of this finding.

## Acknowledgments

We would like to thank staffs of hemodialysis section.

## Authors’ contributions

HN designed and performed the research. MRK analyzed data and wrote some parts of paper. MRK reviewed the draft, too. HN prepared the final draft.

## Conflict of interests

The author declared no competing interests.

## Ethical considerations

Ethical issues (including plagiarism, informed consent, misconduct, double publication and redundancy) have been completely observed by the authors.

## Funding/Support

None.
